# Evidence of Transmission Capability in UK *Culex pipiens* for Japanese Encephalitis Virus (JEV) Genotype I and Potential Impact of Climate Change

**DOI:** 10.3390/v17070869

**Published:** 2025-06-20

**Authors:** Luis M. Hernández-Triana, Sanam Sewgobind, Insiyah Parekh, Nicholas Johnson, Karen L. Mansfield

**Affiliations:** Animal and Plant Health Agency, Addlestone, Surrey KT153NB, UK; sanam.sewgobind@apha.gov.uk (S.S.); nick.johnson@apha.gov.uk (N.J.); karen.mansfield@apha.gov.uk (K.L.M.)

**Keywords:** mosquito, Japanese encephalitis, zoonosis, vector competence, emerging infectious disease

## Abstract

Japanese encephalitis virus (JEV) is a mosquito-borne orthoflavivirus and a major cause of human encephalitis throughout Asia, although it is currently not reported in Europe. To assess the potential impact of climate change, such as increased temperatures, and the potential for native *Cx. pipiens* to transmit JEV genotype I in the United Kingdom (UK), we have investigated vector competence at two different temperatures. *Culex pipiens* f. *pipiens* were provided a bloodmeal containing JEV genotype I at 7.8 × 10^8^ PFU/mL. Mosquitoes were maintained for 14 days at 21 °C or 25 °C, and rates of infection, dissemination, and transmission potential were assessed. There was no evidence for virus infection, dissemination, or potential for transmission at 21 °C. However, at 25 °C, virus infection was detected in 5 of 36 mosquitoes (13.9%). Of these, JEV disseminated to legs and wings in three specimens (3/5) and viral RNA was detected in saliva from one specimen (1/3). These data indicate that at elevated temperatures of 25 °C, UK *Cx. pipiens* f. *pipiens* could transmit JEV genotype 1.

## 1. Introduction

Japanese encephalitis virus (JEV) (Family Flaviviridae/Genus *Orthoflavivirus*) is the principal cause of viral encephalitis in Southeast Asia [1,2]. In nature, the virus is maintained in an enzootic cycle involving mosquito vectors and birds, and infection studies have shown that domestic poultry and pigs may also act as JEV reservoirs. Humans and other mammals such as horses are susceptible to spillover infections but are considered dead-end hosts [1,2].

JEV is currently recognized to consist of five genotypes (GI–GV), determined through genetic analysis and each with differences in their geographical distribution [3]. Genotype III was historically considered to be the most common genotype, with widespread detection in mainland areas of Asia and a single case in Angola [4]. However, JEV genotype I has been reported in mainland China and has gradually expanded its geographical range to include vast areas of South, Central, and East Asia, displacing genotype III to become the dominant genotype in these regions [3]. More recently, an introduction of JEV genotype IV into northern Australia was followed by a subsequent outbreak in eastern Australia [5], providing further evidence for the expansion of JEV into new areas in the country.

In Europe, there have been no cases of Japanese encephalitis in humans or the isolation of live virus in native mosquito populations or wildlife. However, the increased average temperatures and flooding events observed in recent years as a result of climate change have enabled European mosquito populations to flourish, increasing the risk for the establishment of previously undetected viruses in the event of a virus incursion [6]. Previous studies have assessed the competence of European mosquito species to vector several JEV genotypes (Table 1), although no study has assessed genotype I. The mosquito species *Cx. pipiens* is widespread throughout Europe, and it is found in all European countries [7]. Previous studies have shown that temperature has a significant effect on vector competence for JEV genotype III in *Cx. pipiens* f. *pipiens* mosquitoes [7,8,9], a species commonly distributed all over the country, highlighting the potential impact in response to climate change. To assess this further, we investigated the vector competence of UK *Cx. pipiens* f. *pipiens* mosquitoes for JEV genotype I at two different temperatures: 21 °C (representative of current average summer temperatures in the UK) and 25 °C (representative of a projected increase in the UK) [10]. Taken together, these data will address the current knowledge gap and provide further evidence for the impact of climate change on the risk to animal and human health of mosquito-borne viruses in Europe.

## 2. Materials and Methods

A colonized line of *Cx. pipiens* f. *pipiens* ‘Caldbeck UK’ (>100 generations, donated by The Pirbright Institute, UK, originating from Caldbeck, Surrey; mosquitoes were subjected to a photo-period of 12:12 [light/dark], and room conditions were maintained at 24 ± 2.5 °C and above 50% humidity) was infected with JEV as previously described [8,9]. Artificial blood feeding was used to expose two groups of female mosquitoes to JEV genotype I (strain UVE/JEV/2009/LA/CNS769), at a titre of 7.8 × 10^8^ plaque-forming units (PFU)/mL in defibrinated horse blood. The two groups of mosquitoes were subsequently maintained at either 21 °C (representative of current average summer temperatures in the UK) or 25 °C (representative of projected increases in average UK summer temperatures) [10]. Uninfected control mosquitoes were mock-infected and assessed in parallel. At 14 days post-infection (dpi), mosquitoes were anesthetized using FlyNap, legs and wings removed, and the proboscis inserted into a pipette tip containing 20 μL Eagle’s Minimum Essential Medium (EMEM) to encourage saliva expectoration for 45 min; salivation was enhanced by administering 10 μL of Pilocarpin on top of the specimen [8,9]. Tissue samples were homogenized in EMEM prior to RNA extraction and molecular analysis. Viral RNA extraction from homogenates and reverse transcription–polymerase chain reaction (RT-qPCR) were performed as previously referenced in [8,9] using the primers Forward-5′ATCTGGTGYGGYAGTCTCA3′, Reverse-5′CGCGTAGATGTTCTCAGCCC3′, and Probe-5′FAM-CGGAACGCGATCCAGGGCAA-TAMRA3′. For the assessment of vector competence (infection, dissemination, and transmission rates) and virus titration, the protocol of [9,15] was followed. Saliva samples were assessed for viable virus through titration on Vero cells following standard techniques [9,15].

## 3. Results

Feeding rates were similar for both experimental groups of *Cx. pipiens* f. *pipiens*; the group maintained at 21 °C demonstrated a blood-feeding rate of 60.5% (26/43), whilst the group maintained at 25 °C had a 51.6% (36/62) blood-feeding rate (Table 2). Some mosquito mortality was observed up to 7 dpi, and then mosquito survival remained relatively stable before declining toward 13–14 dpi, which is standard in mosquito vector competence experiments.

As expected, there was no detection of viral RNA in any of the tissues taken from uninfected control mosquitoes at 14 dpi. Similarly, for female mosquitoes infected with JEV and maintained at 21 °C, there was no evidence of virus infection, dissemination, or transmission potential at 14 dpi. However, in female mosquitoes infected with JEV and maintained at 25 °C, virus infection was detected in 13.9% (5/36) of specimens, confirmed through molecular detection of viral RNA by RT-PCR (Table 3). Of the five infected specimens, evidence for virus dissemination to legs and wings was detected in 60.0% (3/5). In addition, viral RNA was detected in 33.3% (1/3) of saliva samples from the three specimens with evidence of dissemination, confirming the potential for onward virus transmission in *Cx. pipiens* f. *pipiens* (Table 3).

Molecular analysis of mosquito bodies yielded *ct* values ranging from 20.3 to 34.4, with the wing/leg Ct ranging from 27.1 to 36.0, and the single Ct value for saliva was 34.6. The molecular results were supported by virus titration data for one mosquito specimen only, suggesting that only low-level infection of mosquitoes occurred. For this single specimen, JEV titres for the mosquito tissues analyzed ranged from 2.6 × 10^1^ PFU/mL (mosquito body) to 7.4 × 10^1^ PFU/mL (legs and wings). Viable virus was undetectable in the saliva sample, despite the detection of viral RNA by RT-PCR.

## 4. Discussion

The predominant JEV genotype has shifted from genotype III to genotype I in many regions of Asia [16]; spread of genotypes of other arboviruses via migrating birds such as West Nile virus and Usutu virus follow similar patterns [17]. The results of this study provide evidence that *Cx. pipiens* f. *pipiens* is susceptible to infection with JEV genotype I at 25 °C. We are aware of some of the limitations of our study caused by using two temperatures that we considered most relevant to the UK; nonetheless, we regard that these temperatures are relevant for other European countries, and thus, the approach might be valid for estimating potential infection of JEV in a local population of *Cx. pipiens*. After fourteen days post-infection, the virus was able to disseminate throughout the mosquito body, with viral RNA detected in expectorated mosquito saliva, suggesting that *Cx. pipiens* f. *pipiens* has the potential to be a competent vector for JEV genotype I under the experimental conditions described here. In comparison, this species was not susceptible to infection at the lower temperature of 21 °C. Our findings agree with previous experiments undertaken with European populations of *Cx. pipiens* f. *pipiens* with other JEV genotypes [11,12]. However, we did not observe marked mortality for mosquitoes incubated at the higher temperature of 25 °C, in contrast to the observations previously reported, where mosquitoes infected with JEV genotype III were incubated at the same temperature [8]. The reasons for this difference are unclear, although it may be associated with virus strain- or genotype-specific differences in pathogenicity for *Cx. pipiens* f. *pipiens* mosquitoes. This phenomenon is reminiscent of what is observed with Dengue virus serotypes, where one serotype can predominate for extended periods before being abruptly replaced by another, often with significant public health consequences see review in [18]. This offers a potential avenue for future research with different JEV genotypes.

These data also suggest that if average temperatures continue to rise in Europe, and in the event of a virus incursion via migratory birds or the movement of infected animals or humans, there is the potential for JEV to become established in native populations of *Cx. pipiens* f. *pipiens*, which would pose a risk to both human and animal health. The host preferences of *Cx. pipiens* range from birds and animals, including humans [19,20], which is important as both forms of *Culex pipiens* are widely spread across the UK and the European mainland and they can readily transmit several arboviruses. Our experimental results showed that only a single mosquito had detectable JEV RNA in its saliva after being exposed to a relatively high virus titre (likely higher than those detected in viremic vertebrate hosts). Therefore, this result does provide an indication that local *Cx. pipiens* might transmit JEV genotype I, but it could be argued that it might not be a highly competent vector. In order to assess these questions, further vector competence experiments varying the titre doses and increasing the total number of infected specimens should be carried out. It is also possible that diurnal temperature variation, particularly the lower temperatures experienced between dusk and dawn, may reduce the vector competence of *Cx. pipiens* for JEV by providing a barrier to transmission [8,12], as evidenced by the lack of infection and transmission observed at the lower temperature of 21 °C in the current study. However, recent experience in Europe has shown the extensive transmission and successful overwintering of the related orthoflaviviruses West Nile virus [17] and Usutu virus [21] following initial incursion, highlighting that barriers to orthoflavivirus transmission within the mosquito can be overcome.

## 5. Conclusions

The competence of UK *Culex pipiens* f. *pipiens* (UK Caldbeck population) to vector JEV genotype I is temperature-dependent. This species is not a competent vector at 21 °C but demonstrates the potential for onward transmission at the higher temperature of 25 °C. These data provide further evidence supporting the potential impact of climate change on the transmission potential of a temperate mosquito species for JEV, and the risk to animal and human health in the UK and Europe.

## Figures and Tables

**Table 1:** Summary of European mosquito vector competence studies on Japanese encephalitis virus.

Mosquito Species	JEV Strain	Genotype	Temperature	Days Post Infection	Infection Rate (%)	References (Country)
*Aedes albopictus*	R-9	III	26 °C	13	65	[11] (France)
	SA-21	III	25 °C	14	17	[9] (Italy, Spain)
	XZ0934 R-9	V	26 °C	13	80	[11] (France)
*Aedes detritus*	Muar	V	23 °C	14	78	[12] (United Kingdom)
*Anopheles plumbeus*	Nakayama	III	25 °C 25 °C/15 °C	14	34.1	[13] (Belgium)
*Culex pipiens*	CN5138-11	II	18 °C	21	100	[12] (United Kingdom)
	XZ0934	III	26 °C	13	85	[11] (France)
		V			100	
	SA-21	III	20 °C	14	70	[8] (United Kingdom)
			25 °C		90	
					37	[11] (Belgium)
	Nakayama	III	25 °C	14	35	
			15–25 °C			
	Nakayama	III	25 °C	14, 21, 28	10	[14] (Sweden)
*Culiseta annulata*	CN5138-11	II	24 °C 18 °C	14 21	20 100	[12] (United Kingdom)

**Table 2:** Blood-feeding rate for female *Cx. pipiens* f. *pipiens* infected with Japanese encephalitis virus genotype I and maintained at 21 °C and 25 °C.

Experimental Group	Total Mosquitoes	Total Blood Fed	% Feeding Rate
21 °C			
Control	10	7	70
JEV-infected	43	26	60.5
25 °C			
Control	10	7	70
JEV-infected	62	36	58.1

**Table 3:** Infection, dissemination, and transmission of Japanese encephalitis virus genotype I in *Culex* f. *pipiens* at 21 °C and 25 °C (percentages shown in parentheses).

		Temperature °C	
	21 °C		25 °C
Infectious bloodmeal titre (PFU/mL)	7.8 × 10^8^		7.8 × 10^8^
Infection (Body)	0/26 (0)		5/36 (13.9)
Dissemination (Legs)	0		3/5 (60.0)
Transmission potential (Saliva)	0		1/3 (33.3)

## Data Availability

Data are available on request from the authors.
